# Persistent Newcastle disease virus infection in bladder cancer cells is associated with putative pro-survival and anti-viral transcriptomic changes

**DOI:** 10.1186/s12885-021-08345-y

**Published:** 2021-05-27

**Authors:** Lee-Chin Chan, Jeevanathan Kalyanasundram, Sze-Wei Leong, Mas Jaffri Masarudin, Abhi Veerakumarasivam, Khatijah Yusoff, Soon-Choy Chan, Suet-Lin Chia

**Affiliations:** 1 Department of Microbiology, Faculty of Biotechnology and Biomolecular Sciences, Universiti Putra Malaysia, UPM, 43400 Serdang, Selangor Darul Ehsan Malaysia; 2 Malaysia Genome Institute, Ministry of Science, Technology and Innovation, Jalan Bangi, 43000 Kajang, Selangor Darul Ehsan Malaysia; 3 Department of Cell and Molecular Biology, Faculty of Biotechnology and Biomolecular Sciences, Universiti Putra Malaysia, 43400 UPM Serdang, Selangor Darul Ehsan Malaysia; 4 UPM-MAKNA Cancer Research Laboratory, Institute of Bioscience, Universiti Putra Malaysia, 43400 UPM Serdang, Selangor Darul Ehsan Malaysia; 5 Department of Biological Sciences, School of Medical and Life Sciences, Sunway University, 47500 Bandar Sunway, Selangor Darul Ehsan Malaysia; 6 Medical Genetics Laboratory, Faculty of Medicine and Health Sciences, Universiti Putra Malaysia, 43400 UPM Serdang, Selangor Darul Ehsan Malaysia; 7 Perdana University School of Liberal Arts, Science and Technology (PUScLST), Perdana University, 50490 Kuala Lumpur, Malaysia

**Keywords:** Newcastle disease virus, Bladder Cancer, Persistent infection, Transcriptome analysis, GSEA, Wnt/β-catenin signalling

## Abstract

**Background:**

Newcastle disease virus (NDV) is an oncolytic virus with excellent selectivity against cancer cells, both in vitro and in vivo. Unfortunately, prolonged in vitro NDV infection results in the development of persistent infection in the cancer cells which are then able to resist NDV-mediated oncolysis. However, the mechanism of persistency of infection remains poorly understood.

**Methods:**

In this study, we established persistently NDV-infected EJ28 bladder cancer cells, designated as EJ28P. Global transcriptomic analysis was subsequently carried out by microarray analysis. Differentially expressed genes (DEGs) between EJ28 and EJ28P cells identified by the edgeR program were further analysed by Gene Set Enrichment Analysis (GSEA) and Ingenuity Pathway Analysis (IPA) analyses. In addition, the microarray data were validated by RT-qPCR.

**Results:**

Persistently NDV-infected EJ28 bladder cancer cells were successfully established and confirmed by flow cytometry. Microarray analysis identified a total of 368 genes as differentially expressed in EJ28P cells when compared to the non-infected EJ28 cells. GSEA revealed that the Wnt/β-catenin and KRAS signalling pathways were upregulated while the TGF-β signalling pathway was downregulated. Findings from this study suggest that the upregulation of genes that are associated with cell growth, pro-survival, and anti-apoptosis may explain the survivability of EJ28P cells and the development of persistent infection of NDV.

**Conclusions:**

This study provides insights into the transcriptomic changes that occur and the specific signalling pathways that are potentially involved in the development and maintenance of NDV persistency of infection in bladder cancer cells. These findings warrant further investigation and is crucial towards the development of effective NDV oncolytic therapy against cancer.

**Supplementary Information:**

The online version contains supplementary material available at 10.1186/s12885-021-08345-y.

## Background

Newcastle disease virus (NDV) is a negative, non-segmented, single-stranded RNA paramyxovirus. Despite being pathogenic against the avian species, NDV only causes pharyngitis, conjunctivitis and mild flu-like symptoms in humans [1]. NDV has been studied extensively in vitro and in vivo for its oncolytic properties against various types of cancers [2–5]. The virus has been shown to selectively target cancer cells while leaving normal cells unharmed [5, 6]. It was postulated that the selectivity of NDV is due to defects in antiviral responses that favour viral replication such as the production of interferons by cancer cells [7, 8]. While NDV mediates oncolysis through the activation of intrinsic and extrinsic apoptosis pathways [9], it can also trigger a long-term adaptive immune response against infected cancer cells [10].

However, persistent infection of NDV has been reported in colorectal cancer cells [11]. Interestingly, the persistently infected colorectal cancer cells were found to harbour viral progenies that produced smaller plaques as compared to the uninfected cancer cells [11]. In a separate study, mutations in the HN and F genes were found in the viral progenies that were isolated from persistently infected ovarian cancer cells, linking hyperfusogenic NDV activity and the development of persistent infection [12]. Pertinently, the persistently infected cancer cells were resistant to NDV-mediated oncolysis [12]. Thus, the successful translation of NDV as an oncolytic viral therapeutic in clinic is dependent on the ability to overcome the potential risk of persistent infection.

Interestingly, not all cancer cells develop persistent infection of NDV [11]. Intrinsic cellular factors are thought to play a crucial role, but they remain poorly understood. In this study, we aimed to identify genes that are associated with persistent infection of NDV in EJ28 bladder cancer cells. By comparing the transcriptomic profiles of persistently infected EJ28 cells and uninfected EJ28 cells, we identified differentially expressed genes (DEGs) and pathways that provide novel insights towards our improved understanding of persistent infection of NDV in bladder cancer cells.

## Methods

### Viruses

The method of NDV propagation was previously described [11, 13]. Briefly, the velogenic strain AF2240 was propagated in 9 day-old embryonated eggs and further purified by using a sucrose gradient of 20% (w/v) to 60% (w/v). NDV stock was quantified using plaque assays as previously described [13]. Briefly, 2 × 10^6^ of SW620 colorectal cancer (CRC) cells were seeded into each well of a 6-well plate and incubated in 5% CO_2_ at 37 °C. Cells were incubated for 48 h to ensure it reached 100% confluence before a plaque assay was performed. The recombinant NDV harbouring the GFP gene, rAF-GFP was generated using reverse genetics. The GFP gene was amplified and inserted into the M/F non-coding region of a full-length cDNA clone of the NDV strain AF2240. The recombinant virus was recovered in BSR T7/5 baby hamster kidney cells stably expressing T7 RNA polymerase and subsequently propagated in 10-day old embryonated eggs. Sequencing of the viral genome confirmed the presence of the GFP gene.

### Establishment of persistently infected EJ28 cells model

Persistent infection of NDV in cancer cells was performed as described by Chia et al. [11]. Briefly, EJ28 bladder cancer cells (1 × 10^6^ cells) were seeded into each well of a 6-well plate. On the following day, the confluent monolayer of cancer cells was washed with 1 × PBS followed by infection with NDV at a multiplicity of infection (MOI) of 1. The plate was incubated for an hour and rocked at every 15 min interval. The cells were then rinsed with 1 × PBS, replenished with fresh maintenance media (MM; DMEM supplemented with 2% foetal bovine serum, FBS) and finally incubated for 96 h in 5% CO_2_ at 37 °C. Micrographs of the infected cells were taken regularly to record the progression. The surviving cancer cells were then rinsed with 1 × PBS and fresh growth medium (GM; DMEM supplemented with 10% FBS) was added to allow the surviving cells to grow. Once the cells grew to confluency, the cells were reinfected again with NDV as described above. This process was repeated for another two times to select for truly persistently infected cancer cells. These persistently infected cells were then designated as EJ28P.

### Annexin V/Propidium iodide assay

Reinfection of the persistently infected EJ28P with rAF-GFP was carried out as described above. The cells were passaged continuously. Several passages (1, 15, 20 and 25) were selected to determine the presence of NDV in the EJ28P-GFP cells. The cells were trypsinised by using 0.25% (w/v) of trypsin-EDTA (Gibco, USA), mixed with 1 × PBS at equal volume and then centrifuged at 1000 rpm (Centrifuge 5424, Eppendoff, Germany). The resulting cell pellet was stained with Alexa Fluor® 647 Annexin V apoptosis detection kit (BioLegend, USA) according to the manufacturer’s protocol. The stained cells were then subjected to flow cytometric analysis (Novocyte, Acea Biosciences, USA). By using the NovoExpress Software, 10,000 single cells were gated, and a graph plotted with the FITC channel set as the X-axis and cell count set as the Y-axis. Uninfected EJ28P cells were used as the negative control.

### RNA extraction and microarray analysis

Total RNA of EJ28 and EJ28P cells were extracted using RNeasy MinElute™ Kit (Qiagen, The Netherlands) according to the manufacturer’s protocol. The RNA quantity and purity were analysed by NanoPhotometer (Implen, Germany). RNA samples with A260/A280 ratio of 2.0 and above were subjected to Bioanalyzer (2100 Expert, Agilent Technologies, USA) analysis to determine the integrity of the extracted RNA. Only RNA samples with an RNA integrity number (RIN) of 8 and above were selected for the subsequent analyses. RNA samples with RIN values lower than 8 were re-purified with the RNeasy MinElute™ Kit (Qiagen, The Netherlands) according to the manufacturer’s protocol. The RNA samples were then diluted to 300 ng/μL and subsequently labelled using TargetAmp™-Nano Labeling Kit for Illumina® Expression BeadChip® (Epicentre, USA). All incubation steps were performed on the Veriti 96-Well Thermal Cycler (Applied Biosystems, USA). Subsequently, the generated biotin-aRNA was purified using the RNeasy MinElute Cleanup Kit (Qiagen, Germany) according to the manufacturer’s protocol. The purified biotin-aRNA was quantified and further diluted to a concentration of 150 ng/μL in a 15 μL solution, followed by analysing it on the Bioanalyzer using the RNA Nano Chip. The RNA samples that passed the QC were run on the HumanHT-12 v4 Expression BeadChip (Illumina, USA). The BeadChip was hybridised for 18 h 24 min at 58 °C, and the detection was carried out using Cy3-Streptavidin (Invitrogen, USA). The hybridised BeadChip microarray was scanned using the iScan System (Illumina, USA). The microarray data were submitted to the GEO database and the accession number is GSE163881.

### Differentially expressed genes (DEG) and gene set enrichment analysis (GSEA)

DEGs between EJ28P and EJ28 obtained from the iScan System was presented as a Volcano plot. The plot was constructed by plotting the –log10 of the *p*-values on the Y-axis and log2 fold change on the X-axis. Subsequently, the edgeR program was used to select genes that had significant changes (*P*adj < 1e-10) with an absolute log2 fold change of 2 (upregulated genes) and − 2 (downregulated genes). GSEA was performed to identify relevant biological significances by using the latest version of GSEA software (4.1.0) downloaded from Broad Institute Gene Set Enrichment Analysis website (www.broad.mit.edu/gsea). The enrichment gene sets used were selected from MSigDB, namely, hallmark (H), curated (C2), oncogenic (C6) and immunologic (C7) gene sets. The phenotype label was persistent infection versus control and the number of permutations was set to 1000. Significance of enrichment magnitude was set at a False Discovery Rate (FDR) of 25% for GSEA.

### Ingenuity pathway analysis (IPA)

DEGs with log2 fold change of > 2 (upregulated genes) and < 2 (downregulated genes) were selected and analysed using IPA (QIAGEN Inc., https://www.qiagenbioinformatics.com/products/ingenuity-pathway-analysis). The software uses a network generation algorithm to segment the network map between molecules into multiple networks. In addition, IPA was also used to compare the relationship among these DEGs to identify key regulators within the network.

### RT-qPCR analysis

The total RNA extracted from EJ28 and EJ28P was converted into cDNA using SensiFAST™ cDNA synthesis kit according to the manufacturer’s protocol (Bioline, United Kingdom). Quantitative reverse transcription PCR (RT-qPCR) analysis was performed in three technical replicates to measure the relative gene expression of five randomly selected DEGs from the microarray dataset. The delta-delta Ct method was used to determine the expression ratio between EJ28P and EJ28 cells, where normalisation was performed using three housekeeping genes, namely, *TBP* (QT00000721), *SDHA* (QT00059486), and *GAPDH* (QT00079247). The five selected DEGs were *BNIP3* (QT00024178), *S100A4* (QT00014259), *DDIAS* (QT02451288), *CASP9* (QT00036267), and *APOBEC3B* (QT00040733). All the primers used in this study were purchased from Qiagen (QuantiTect@ Primer Assay).

## Results

### Establishment of NDV-persistently infected EJ28 (EJ28P) cells

**Fig. 1:**
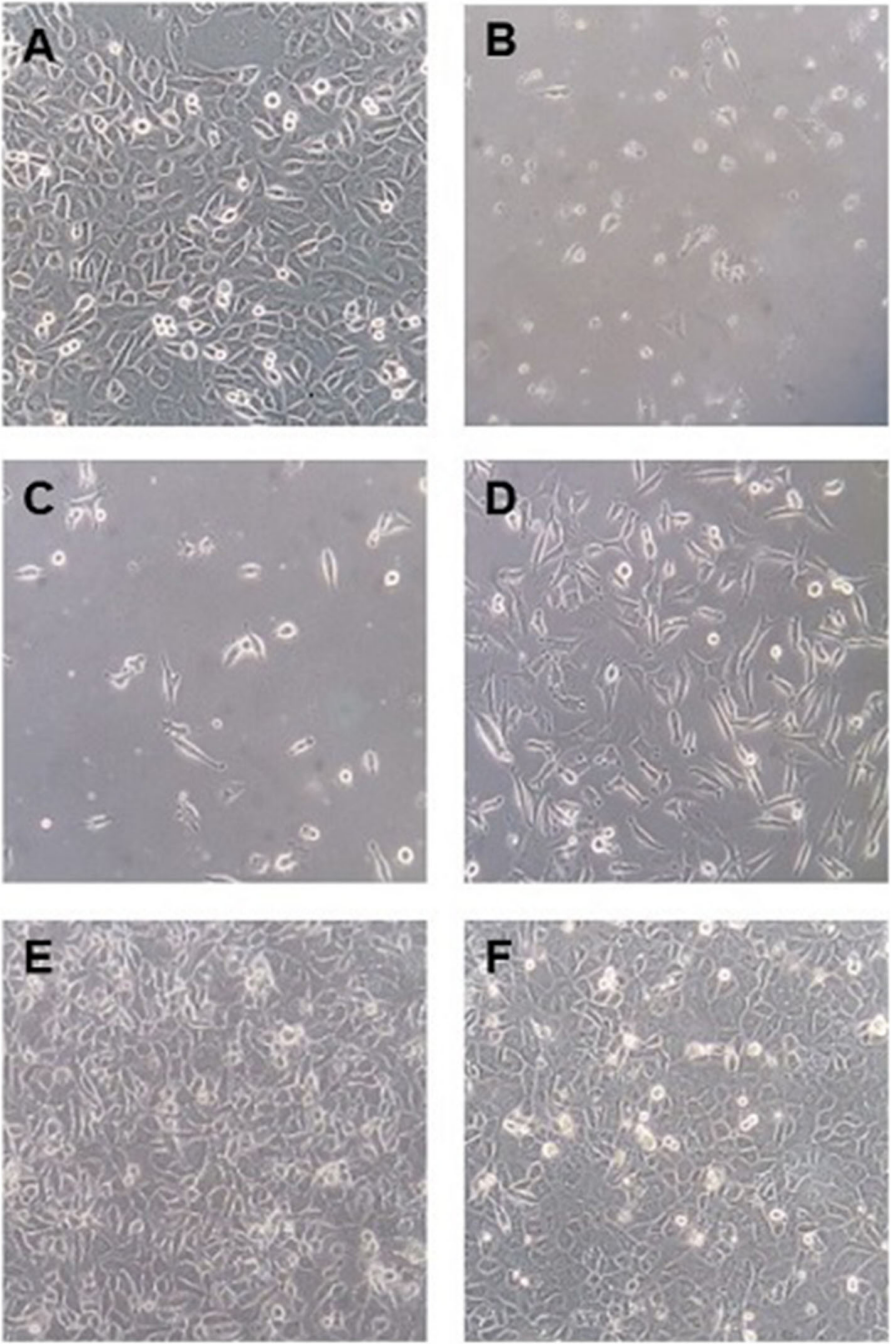
Establishment of NDV-persistently infected EJ28P cells. EJ28P cells were established after three NDV infections at MOI of 1. A subpopulation of cells that survived the first infection were allowed to grow to confluency and were reinfected twice with NDV. (**A**) Day 1 (first infection), (**B**) Day 5, (**C**) Day 12, (**D**) Day 17 (second infection), (**E**) Day 21 (third infection) and (F) Day 27

When the EJ28 bladder cancer cells were first infected with the NDV strain AF2240 (MOI of 1), majority of the infected cells died. The surviving subpopulation of cells persisted in a state of slow growth for about two weeks (Fig. 1). Once the surviving subpopulation reached confluency on day 17, the cells were reinfected with NDV (MOI of 1) and subsequently reinfected again on day 21. No gross cytopathic effects were observed upon NDV reinfection. The surviving cells from the first NDV infection appeared to be resistant to NDV-mediated oncolysis. However, these cells could either have been a result of the preferential selection of inherently resistant subpopulation of cells during the first infection or the acquiring of persistency of infection in culture post-infection; or a combination of both. These persistently NDV-infected cells were designated as EJ28P.

### EJ28P cells harbour NDV over multiple passages and are resistant towards NDV-mediated oncolysis

**Fig. 2:**
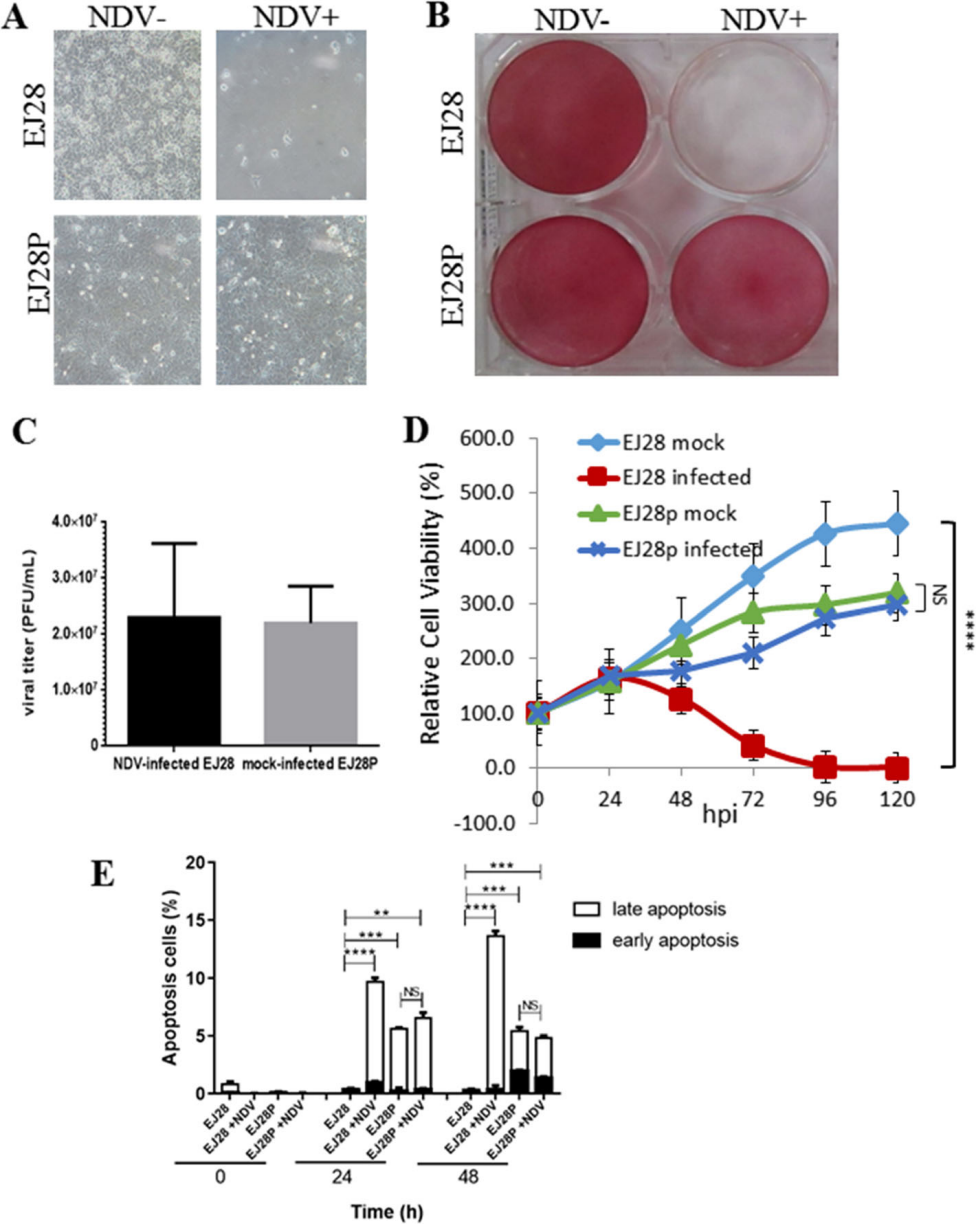
Confirmation of NDV persistently infected EJ28P cells. (**A**) Microscopic image and (**B**) neutral red staining of mock- and NDV-infected EJ28P and EJ28 cells (MOI of 1) at 120 hpi. (**C**) Virus titre of the culture supernatant collected from EJ28P and NDV-infected EJ28 (positive control) cells. (**D**) Relative viability of mock- and NDV-infected EJ28P and EJ28 cells (MOI = 1) from 0 hpi to120 hpi. (**E**) Apoptosis analysis of NDV-infected and mock-infected EJ28 and EJ28P cells stained with Annexin V and propidium iodide, followed by flow cytometric analysis. Percentage of early and late apoptotic cells are presented in a bar graph. All data were presented as mean of biological triplicate readings. Value with ****, ***, **, and NS indicate a *P* value of < 0.0001, < 0.001, < 0.01, and not significant, respectively

In order to compare the NDV cytotoxicity against EJ28P and EJ28 cells, both cells were infected with NDV (MOI = 1) and observed under the microscope at 120 hpi. As previously observed during the establishment of EJ28P, the EJ28 cells experienced cytopathic effects with only a few surviving cells. The EJ28P, on the other hand, did not display any gross NDV-induced cytopathic effects (Fig. 2a). The viable cells post-NDV infection was qualitatively determined by neutral red staining (Fig. 2b). The wells containing EJ28 cells infected with NDV appeared transparent with only a few adherent cells stained with neutral red whereas the well containing EJ28P cells infected with NDV appeared to be completely stained in red; much like the wells containing the mock-infected cells. The virus titre in these EJ28P cells were determined via plaque assay (Fig. 2c). The EJ28P were found to produce viral progenies up to 2.2 × 10^7^ PFU/mL comparable to that of the infected EJ28. Interestingly, the plaques produced by NDV in EJ28P cells were smaller in size as compared to that produced by NDV during the 1st infection of EJ28 cells. (see Additional file 1: Fig. S1).

The cell viability of mock- and NDV-infected EJ28 and EJ28P cells from 0 hpi to 120 hpi was also analysed using trypan blue exclusion test (Fig. 2d). At 24 hpi, the relative cell viabilities were similar across all four cells. However, significant differences in relative cell viability values were observed in the subsequent time points. At 120 hpi, there were less than 1% viable NDV-infected EJ28 cells. In contrast, the mock-infected EJ28 cells had quadrupled while the mock- and NDV-infected EJ28P cells had tripled in that same 5-day period. Although the relative cell viability was lower than that of the parental cells, EJ28P cells appear to be resistant towards NDV-mediated oncolysis. To further confirm the presence of NDV in EJ28P, we reinfected these cells using a recombinant NDV strain AF2240 that harbours the green fluorescent protein (GFP) gene (rAF-GFP) at MOI of 1. Subsequent observation under a fluorescence microscope showed that GFP was expressed in the EJ28P cells (see Additional file 1: Fig. S2). The GFP signal was detectable in the EJ28P cells even after 25 passages. These findings suggest that persistently infected EJ28P cells are susceptible to NDV infection and was able to continuously produce viral proteins through multiple cell passages.

Annexin V/Propidium Iodide assay was also conducted on the mock- and NDV-infected EJ28 and EJ28P cells and analysed by flow cytometry (see Additional file 1: Fig. S3). At both time points (24 hpi and 48 hpi), there were no statistically significant differences in the percentages of apoptotic cells (late or early) between mock- and NDV-infected EJ28P cells. This further validates that NDV reinfection has limited oncolytic effect on persistently infected cells (Fig. 2e). Nevertheless, it is interesting to note that although no gross cytopathic activity was observed in the earlier assays, there were a relatively small percentage (~ 5%) of apoptotic cells in both mock- and NDV-infected EJ28P cells. Similarly, apoptotic cells were detected in persistently infected Hep2 cells in a previous study [14].

### A panel of 20 gene expression signatures provides accurate discriminatory power to distinguish NDV-persistently infected cells from their parental cells

**Fig. 3:**
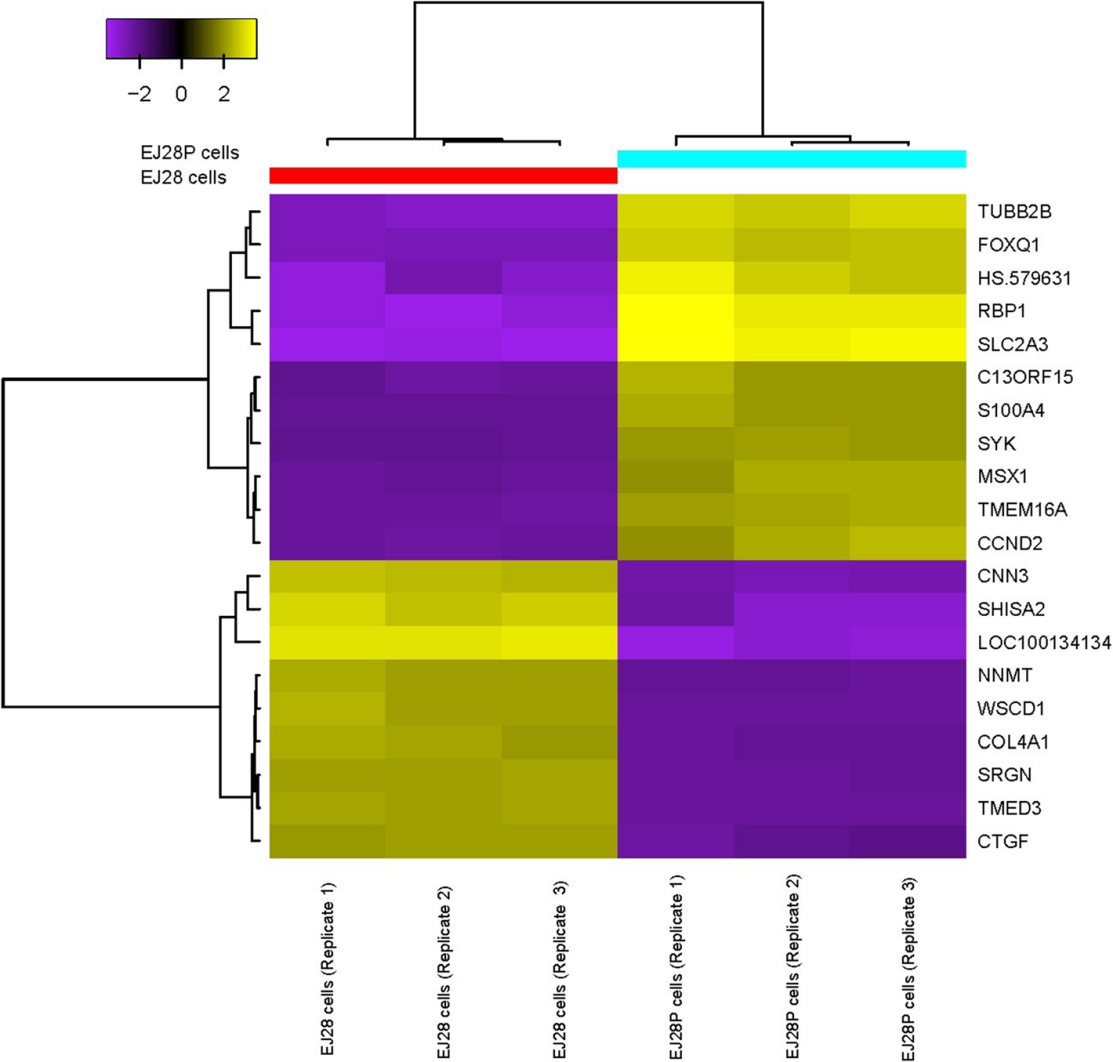
Heatmap of differential gene expression between EJ28 and EJ28P cells. The gene expression profiles of 20 genes provided sufficient discriminatory power to accurately separate EJ28 and EJ28P cells into 2 distinct clusters representing bladder cancer cells and persistently infected bladder cancer cells. Downregulated genes are represented in purple (expression value < 0) while upregulated genes are represented in yellow (expression value > 0)

Microarray analysis of the global gene expression identified a total of 368 genes that were significantly differentially expressed in EJ28P cells as compared to EJ28 cells. Of these, 229 genes were upregulated and 139 genes were downregulated in EJ28P cells (see Additional file 2: Table S1). A heatmap (Fig. 3) was generated using 20 DEGs, which provided sufficient discriminatory power to separate both EJ28 and EJ28P cells into two distinct hierarchical clusters, which are the bladder cancer cells and the persistently infected bladder cancer cells clusters, respectively. Although GSEA identified more than 20 genes that were distinctively expressed between these two clusters of cells (see Additional file 3: Fig. S4), only 20 genes were needed to establish a panel of genes to distinguish persistently infected EJ28P cells from non-infected EJ28 cells.

It was found that regulators of cell cycle and cell proliferation such as *S100A4* [15, 16], *CCND2* [17], *C13ORF15 (RGCC)* [18, 19]*,* and *SYK* [20, 21] and genes associated with glucose or ion transport such as *SLC2A3* [22–24] and *TMEM16A (ANO1)* [25, 26] were upregulated in the persistently infected bladder cancer cells. While *CNN3*, *SHISA2*, *TMED3, SRGN* were downregulated in persistently infected bladder cancer cells. It is worth mentioning that *SHISA2* is a negative regulator of Wnt/β-catenin signalling pathway [27–29] while *SRGN* is a mediator of granule-mediated apoptosis [30–32]. The details of these 20 significant DEGs are shown in Additional file 3: Table S2.

### GSEA identifies pathways associated with cell survival, cell growth and differentiation as upregulated in EJ28P

**Fig. 4:**
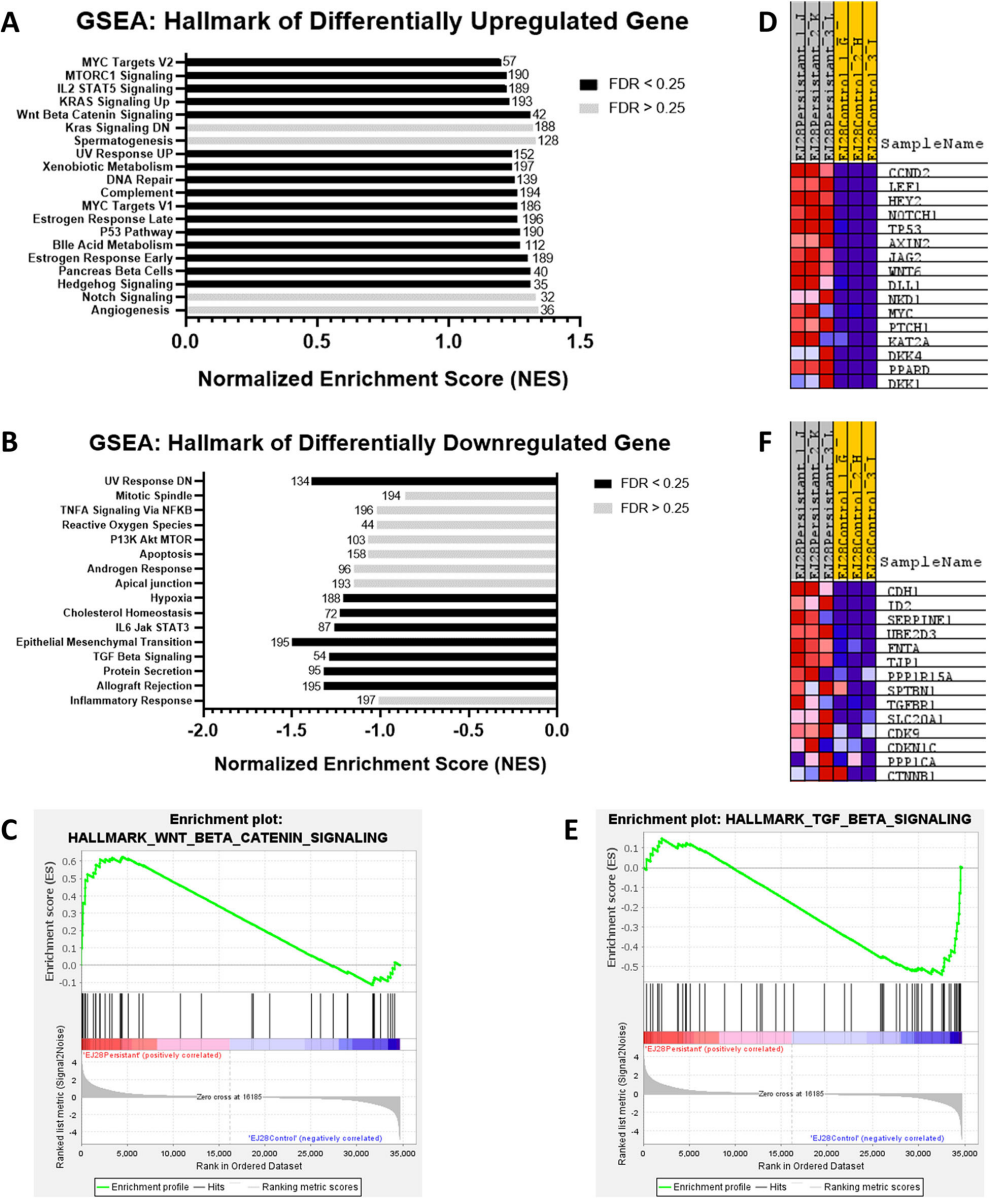
GSEA reveals activation of cell proliferation and cell growth signalling pathways in EJ28P. GSEA was performed using the hallmark genes set from MSigDB with (**A**) upregulated genes and (**B**) downregulated genes. Enriched pathways with FDR < 0.25 are shown in black while FDR > 0.25 are shown in grey. GSEA enrichment plots for (**C**) Hallmark-Wnt/β-catenin Signalling, (**D**) Hallmark-TGF-β Signalling. (**E**) Heatmap of enriched genes in the Wnt/β-catenin signalling pathway. (**F**) Heatmap of enriched genes in the TGF-β signalling pathway

The microarray data analysis with GSEA based on MSigDB hallmark gene sets revealed the most enriched pathways in EJ28P cells Fig. 4 (4A and 4B). Among these pathways, Wnt/β-catenin signalling pathway (Fig. 4c) was the most induced gene set in the hallmark with an NES of 1.31. Figure 4d shows the list of genes associated with the canonical Wnt/β-catenin signalling pathway such as *CCND2*, *AXIN2*, *LEF1, NKD1,* and *NOTCH1* that were found to be upregulated in EJ28P. Meanwhile, some downregulated DEGs were found to be enriched in the TGF-β signalling pathway (Fig. 4e & f). GSEA showed that KRAS signalling was also enriched in EJ28P cells (see details in Additional file 3: Table S3). DEGs that are involved in the inhibition of apoptosis such as *TNFRSF1B, TMEM158,* and *FGF9* were found to be enriched in this pathway, [33–37].

**Fig. 5:**
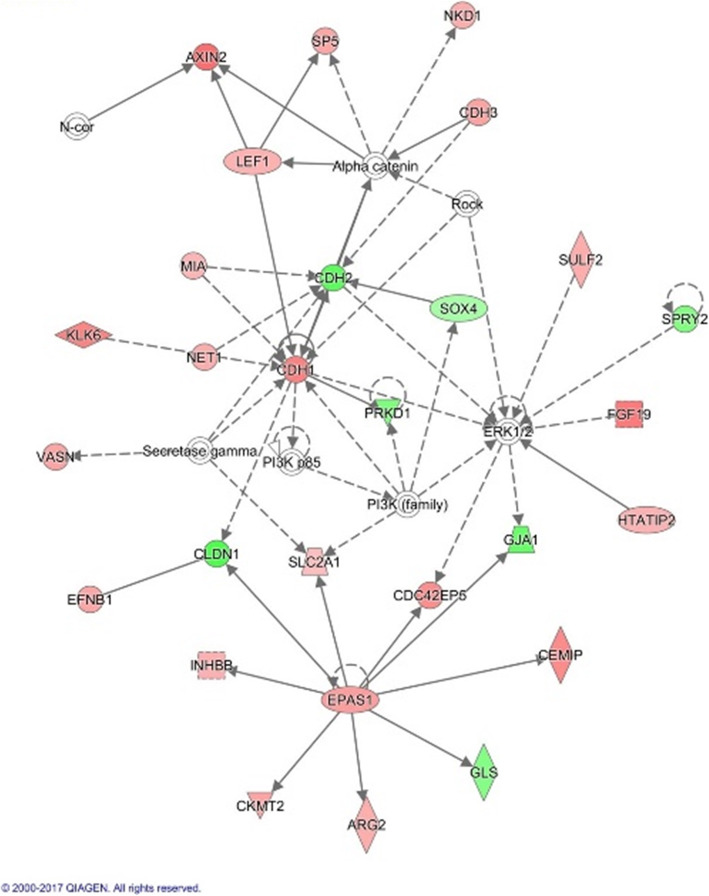
Network of gene interactions identified by IPA. The network is displayed graphically as nodes (genes) and edges (the biological relationships between nodes). The network shows that *EPAS1* is an important regulator gene that controls the expression of multiple genes. Most of the genes within the network are associated with the Wnt/β-catenin signalling pathway. Nodes coloured in red indicates upregulated expression while nodes coloured in green indicates downregulated expression. (QIAGEN Inc., https://www.qiagenbioinformatics.com/products/ingenuity-pathway-analysis)

IPA was employed to search for potential interactions between the DEGs that were identified from the microarray data. Corresponding to the GSEA findings, one of the identified networks (Fig. 5) revealed several key genes associated with the Wnt/β-catenin signalling pathway such as *CDH1, EPAS1, AXIN2, LEF1, NKD1.* The network showed that *EPAS1* was an important regulator gene that controls multiple genes while *CDH1* has multiple interactions with other genes.

**Fig. 6:**
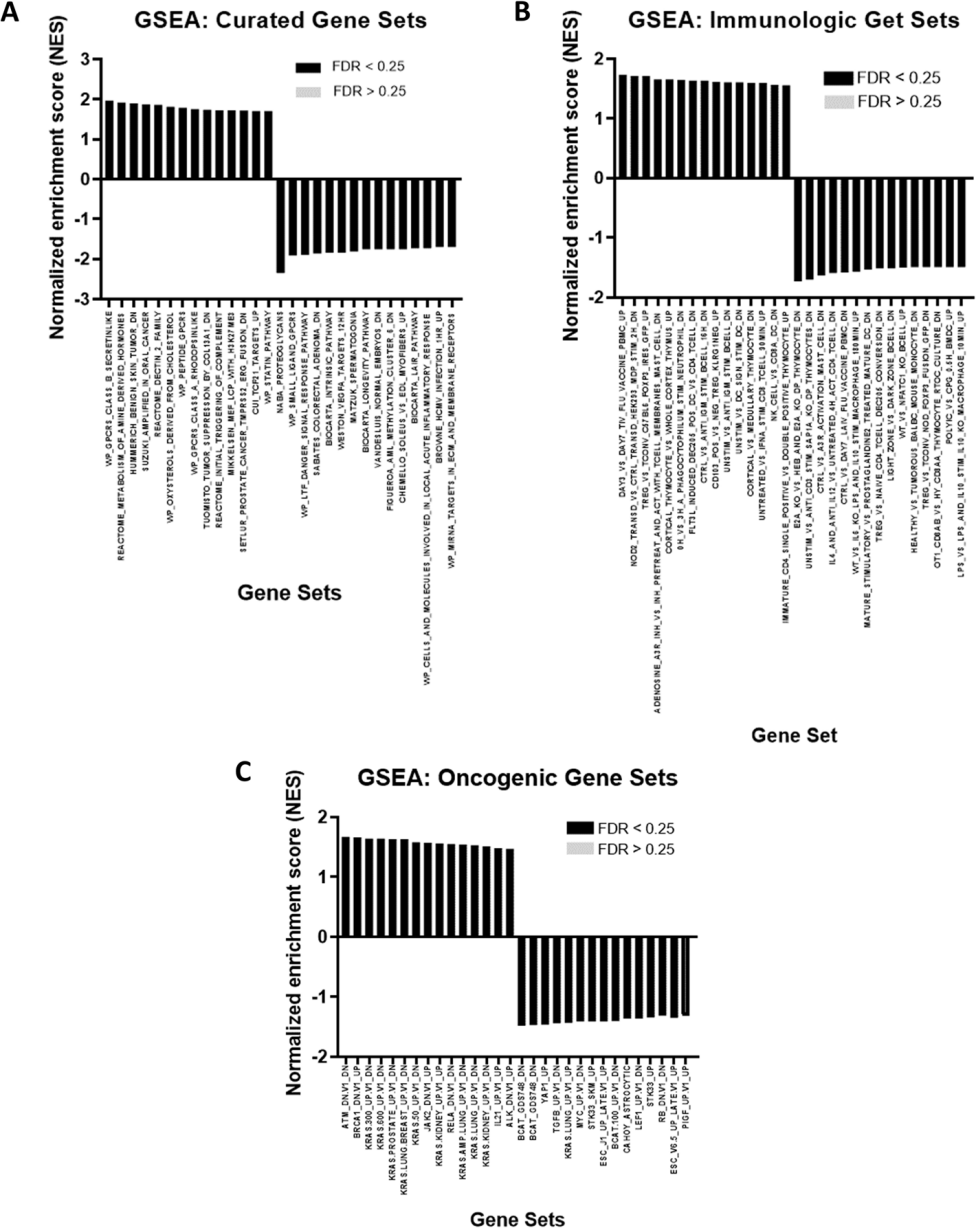
Enrichment analysis by GSEA using different gene set collection. (**A**) GSEA using the Curated Gene Set collection. (**B**) GSEA using the Immunologic Gene Sets collection. (**C**) GSEA using the Oncogenic Gene Sets collection. All the significantly enriched pathways are presented in black (FDR < 0.25) or grey (FDR < 0.25)

Subsequently, GSEA was performed using curated (Fig. 6a), immunologic (Fig. 6b), and oncogenic (Fig. 6c) gene sets collection. The curated gene sets revealed that the top 3 significantly enriched pathways were GPCRs class B Secretin-like (NES = 1.97; FDR = 0.186), metabolism of amine-derived hormones (NES = 1.92; FDR = 0.155) and benign skin tumour (NES = 1.90; FDR = 0.123). GSEA analysis of 189 oncogenic gene sets identified the signature characteristics of genes downregulated in HEK293 cells upon knockdown of ATM (NES = 1.67; FDR = 0.062), genes upregulated in MCF10A cells (breast cancer) upon knockdown of *BRCA1* (NES = 1.66; FDR =0.062); and genes downregulated in epithelial cells expressing the mutated form of *KRAS* (NES = 1.64; FDR = 0.062) to be the top 3 significantly enriched in EJ28P cells. The immunologic gene sets revealed the top 3 significantly enriched immune-related gene sets were for the signature characteristics of genes upregulated in response to trivalent inactivated influenza (TIV) vaccination (NES = 1.73; FDR =0.055); genes downregulated in HEK293 cells at 2 h after stimulation by muramyl dipeptide (NES = 1.71; FDR =0.090); genes upregulated in regulatory T cells due to altered function of *FOXP3* (NES = 1.71; FDR =0.078).

### Validation of microarray data by RT-qPCR

**Fig. 7:**
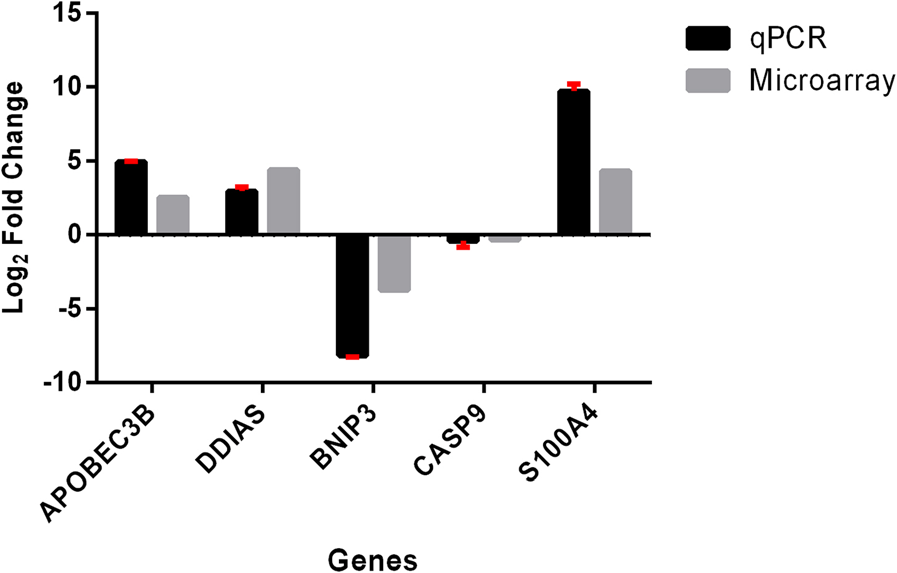
Validation of microarray data by RT-qPCR analysis. The relative expression of the selected DEGs quantified by RT-qPCR and microarray are presented as log2 fold change. The expression pattern was similar. Both techniques found that three DEGs (*APOBEC3B*, *DDIAS*, and *S100A4*) were upregulated while two DEGs were downregulated (*BNIP3* and *CASP9*). All data were presented as mean ± standard deviation (*n* = 3)

In order to validate the microarray results, five DEGs were randomly selected and subjected to RT-qPCR analysis. Based on the similar expression patterns, the log_2_ fold change values derived from the RT-qPCR data validated the DEGs that were identified from the microarray-based profiling exercise. (Fig. 7).

## Discussion

Despite NDV’s ability to lyse cancer cells, some cancer cells including colorectal cancer and ovarian cancer are able to resist viral-mediated oncolysis and eventually become persistently infected with NDV. The intrinsic mechanism underlying persistent infection of NDV remains elusive. In this study, we established persistently NDV-infected bladder cancer cells, EJ28P, by challenging EJ28 bladder cancer cells with NDV infection over a period of 2 weeks. Eventually, there was a subpopulation of cells that survived the viral infection and grew uninterruptedly despite exposure to a second and third infection. These findings are similar to that reported in a study involving colorectal cancer cells [11].. These cells were verified to be persistently infected by analysing the cell viability, plaque assay of spent medium, and Annexin V/Propidium Iodide assay. The overall characteristics of EJ28P were similar to other NDV persistently infected cancer cells that have been reported previously [11] such as (i) resistance to NDV superinfection and (ii) continuously producing viral progenies. Nevertheless, a small population of early apoptotic cells were observed in mock-infected EJ28P cells as compared to the mock-infected EJ28 cells. Similar observation was also reported by Fox & Parks (2018), who concluded that the persistently infected cells have higher basal levels of cellular stress that contributes to the relatively small percentage of cell death [14]. To pinpoint the possible underlying mechanism(s) associated with persistent NDV infection, we performed transcriptome analysis to identify significant genes and pathways that have been altered in NDV persistently infected EJ28P as compared to EJ28 cells.

GSEA was used to identify the hallmark gene sets that have been differentially regulated in EJ28P cells. Gene sets that were upregulated included Wnt/β-catenin signalling, hedgehog signalling, oestrogen response early and late, and MYC target V1 and V2. All these gene sets are involved in the regulation of cell cycle, cell proliferation and cell growth. Interestingly, a previous study on the mechanism of NDV infection showed that NDV mediates oncolysis via the downregulation of Wnt/β-catenin signalling pathway to promote apoptosis and inhibit cell migration [38]. This pathway could be dysregulated by viral-host interaction in order to establish and maintain viral latency in host cells [39]. Hence, we hypothesised that Wnt/β-catenin signalling pathway could be involved in the persistent infection of NDV in EJ28 cells.

NDV is well known for its ability to induce both extrinsic and intrinsic apoptotic signalling in cancer cells [9]. However, the ability of EJ28P to survive NDV-mediated oncolysis suggests that the anti-apoptosis or pro-survival signalling could be upregulated. The transcriptome analysis revealed several genes, which are associated with the regulation of cell survival such as *S100A4* and *SYK,* both of which were upregulated in EJ28P. Incidentally, S100A4 expression is positively regulated by the Wnt/β-catenin signalling pathway [40] to protect cells from pro-apoptotic stimuli [41, 42]. It was reported that the knockdown of *S100A4* decreased cellular proliferation and promoted apoptosis [42]. Meanwhile, the *SYK* gene was reported to provide pro-survival signals [43] whereas the inhibition of its protein expression resulted in apoptosis and the suppression of cellular proliferation [44].

TGF-β signalling pathway have been shown to be upregulated by many viruses, such as hepatitis B virus, Influenza A virus and lymphocytic choriomeningitis virus, during infection [45–48]. Nevertheless, GSEA analysis showed that this pathway was downregulated in EJ28P cells. The downregulation of this pathway is hypothesised to hamper successful viral replication in the host cells. However, our study showed that the EJ28P cells were persistently infected by NDV and the cells continued to produce viral progenies. This suggests that TGF-β signalling pathway may not play an important role in the persistent infection of NDV in EJ28P. Bottler et al. (2012) also showed that TGF-β blockade failed to control the establishment of persistent virus infection [45].

GSEA also revealed that DEGs associated with KRAS signalling were enriched in EJ28P cells. Activation of the proto-oncogene KRAS mutation pathway is common in cancer cells and it is responsible for promoting apoptosis inhibition, migration and proliferation in many cancer cells [49–51]. In addition, KRAS signalling enhances the Wnt/β-catenin signalling resulting in tumour multiplicity and progression [52]. A close-up analysis on these DEGs showed that several negative regulators of apoptosis were upregulated in EJ28P cells, including *TNFRSF1B, TMEM158,* and *FGF9*. Several studies reported that TNFRSF1B could induce pro-survival pathways and protect cells from TNF-induced apoptosis [33, 34, 53, 54]. On the other hand, in vitro siRNA knockdown of *TMEM158* have resulted in the inhibition of cell proliferation and increased apoptosis in cancer cells [35, 36]. Increased levels of *FGF9* expression via transient transfection has shown to decrease cisplatin-induced cellular apoptosis while siRNA knockdown of *FGF9* increased cisplatin-induced cellular apoptosis [55].

It was also revealed that cell proliferation and cell growth associated genes such as *CCND2* and *CL13ORF15* (*RGCC*) were upregulated in EJ28P cells. CCND2 plays a critical role in cell cycle regulation where it was reported that overexpression of CCND2 in cancer cells is associated with enhanced cell proliferation and aggressiveness [56]. In addition, CL13ORF15 (RGCC) was found to modulate the cell cycle and induce mitosis [57]. The upregulation of glucose transporter protein genes such as *SLC2A3* and *TEMEM16A* (*ANO1*) also suggest that the cells require large amounts of energy in order to sustain cellular metabolism that is required for cellular proliferation and growth due to the upregulation of the Wnt/β-catenin signalling pathway. In a nutshell, the transcriptome analysis and GSEA collectively support the postulate that Wnt/β-catenin signalling pathway is involved in persistent infection of NDV in bladder cancer cells via the modulation of cellular survival, proliferation, and anti-apoptosis.

## Conclusions

This study established persistent infection of NDV in bladder cancer cells and identified putative genes and pathways that are associated with persistent infection. It provides a snapshot of the cohesive transcriptomic dysregulation that occurs during persistent infection of NDV in bladder cancer cells (i.e. EJ28P). The biological significance of the Wnt/β-catenin signalling pathway in conferring and maintaining the persistent infection warrant further investigation.

## Supplementary Information

**Additional file 1: Figure S1**: Comparison of virus titres between EJ28 and EJ28P; **Figure S2**: GFP expression of rAF-GFP-infected EJ28P; **Figure S3**: Parental EJ28 and EJ28P infected or mock-infected with NDV labelled with annexin V and PI;

**Additional file 2: Table S1**: Differently expressed genes with Padj < 1–10 and absolute LOG2 fold change of 2 in expression levels between EJ28P and EJ28

**Additional file 3: Figure S4**: Gene markers for the EJ28P versus EJ28 comparison; **Table S2**: Details of top 20 significant DEGs between EJ28P and EJ28; **Table S3**: Gene set of HALLMARK_KRAS_SIGNALING_UP enriched in EJ28P

## Data Availability

The datasets used and/or analysed during the current study are available from the corresponding author on reasonable request.

## Abbreviations

NDV: Newcastle disease virus
GSEA: Gene set enrichment analysis
RT-qPCR: Quantitative reverse transcription reaction chain
KEGG: Kyoto Encyclopedia of Genes and Genomes
DEG: Differentially expressed gene
TGF-β: Transforming growth factor beta
TNF: tumour necrosis factor
PBS: Phosphate-buffered saline
CRC: Colorectal cancer
GFP: Green fluorescent protein
MM: Maintenance medium
GM: Growth medium
DMEM: ‘s Modified Eagle Medium
FBS: Foetal bovine serum
CO_2_: Carbon dioxide
EDTA: Acid
RIN: RNA integrity number
PFU: Plaque forming unit
hpi: Hour post infection
rAF-GFP: Recombinant AF2240 GFP gene
IPA: Ingenuity Pathway Analysis
SYK: Spleen associated tyrosine kinase
