# Supplementary material for: Persistent Newcastle disease virus infection in bladder cancer cells is associated with putative pro-survival and anti-viral transcriptomic changes
Source: BMC Cancer. 2021 May 27;21:625. doi: 10.1186/s12885-021-08345-y (PMC8161962; doi:10.1186/s12885-021-08345-y)
Supplement: Supplementary file 1 — Additional file 1: Figure S1: Comparison of virus titres between EJ28 and EJ28P; Figure S2: GFP expression of rAF-GFP-infected EJ28P; Figure S3: Parental EJ28 and EJ28P infected or mock-infected with NDV labelled with annexin V and PI; [file 12885_2021_8345_MOESM1_ESM.docx]

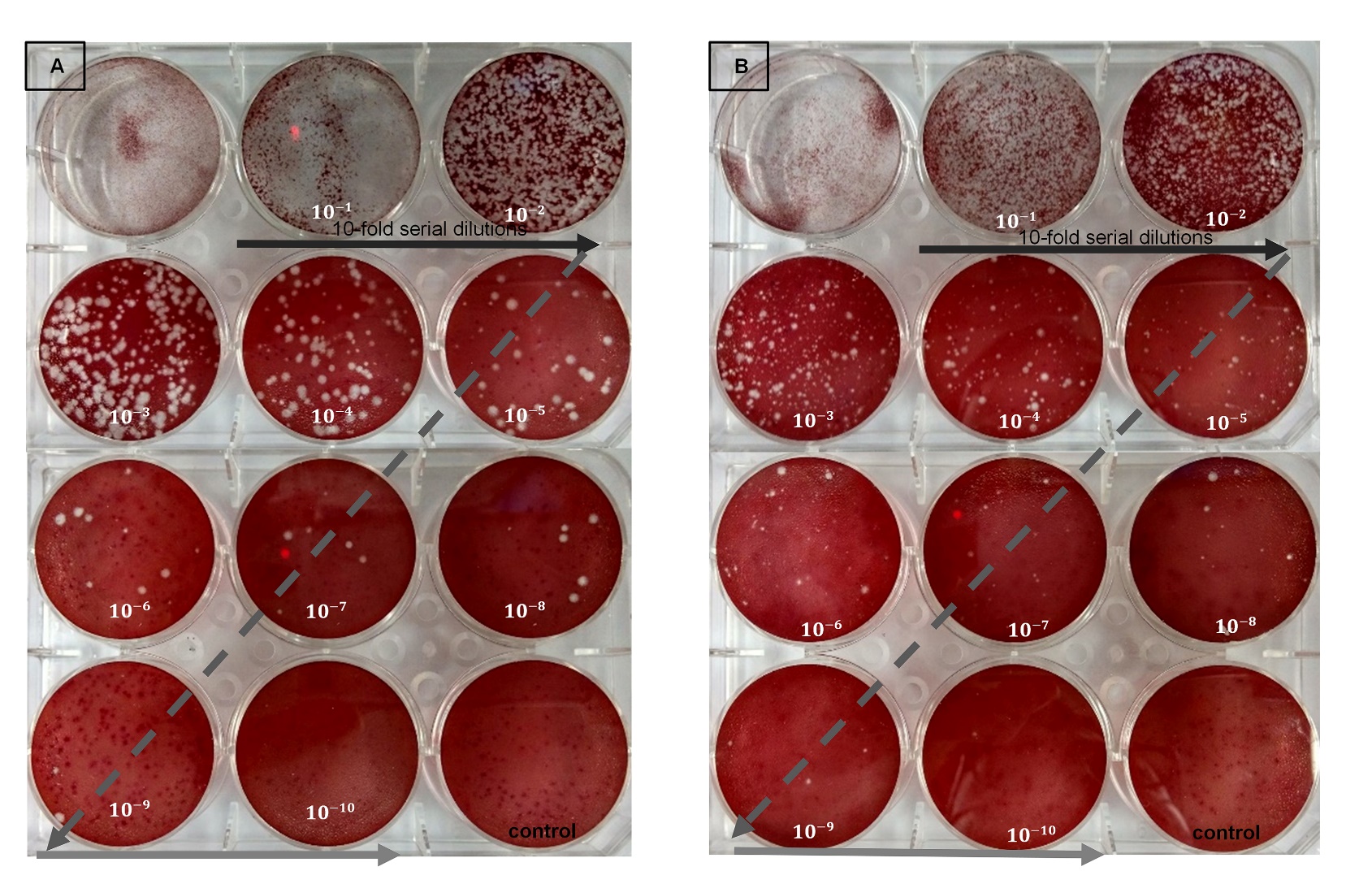


**Figure S1: Comparison of virus titres between EJ28 and EJ28P.** Virus titre of (A) NDV-infected parental EJ28 at MOI=1 and (B) mock-infected EJ28P were determined by their supernatant at 48 hpi using plaque assay. Plaque formation was visualised by neutral red after 120 hours post infection (hpi). Data shown is one representative of biological triplicate results.


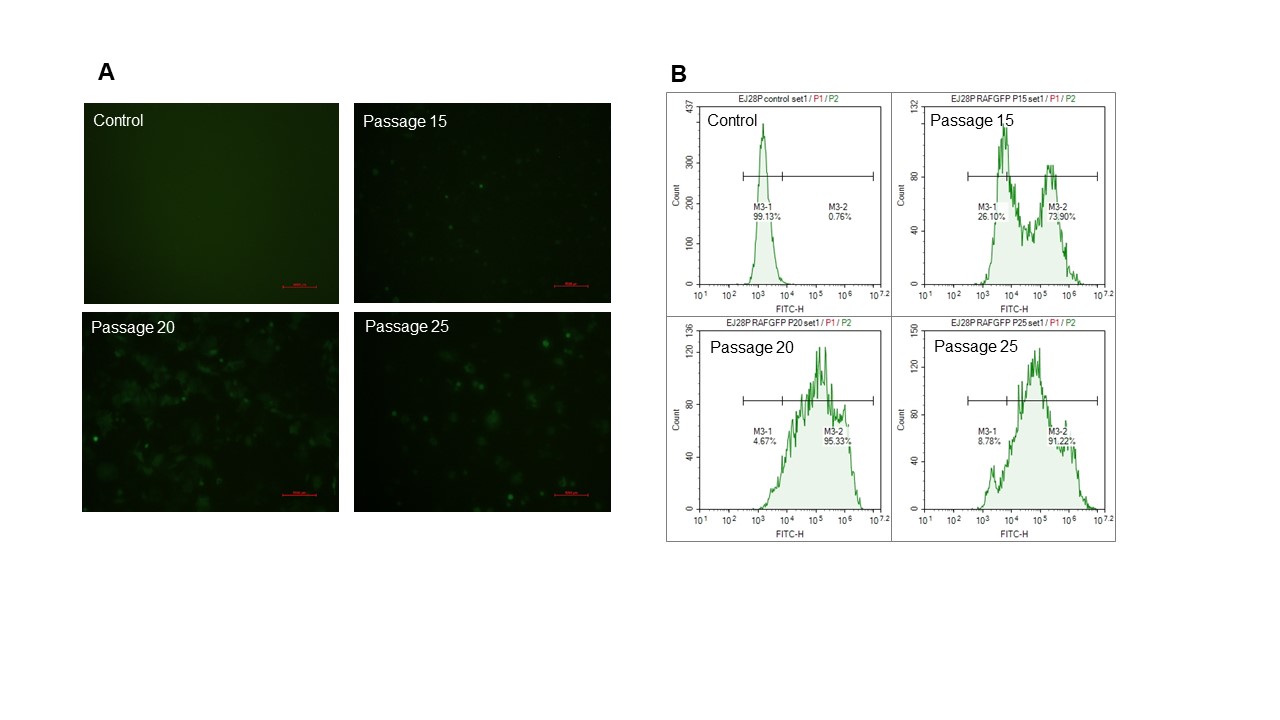


**Figure S2**: **GFP expression of rAF-GFP-infected EJ28P.** (A) EJ28P was infected with rAF-GFP at MOI=1 and maintained in T-75 flask. At every passage, the GFP signal was detected by using the fluorescence microscope. The fluorescence was still detectable even at passage 25. (B) Flow cytometry of GFP expression level at passage 15, 20 and 25.

**
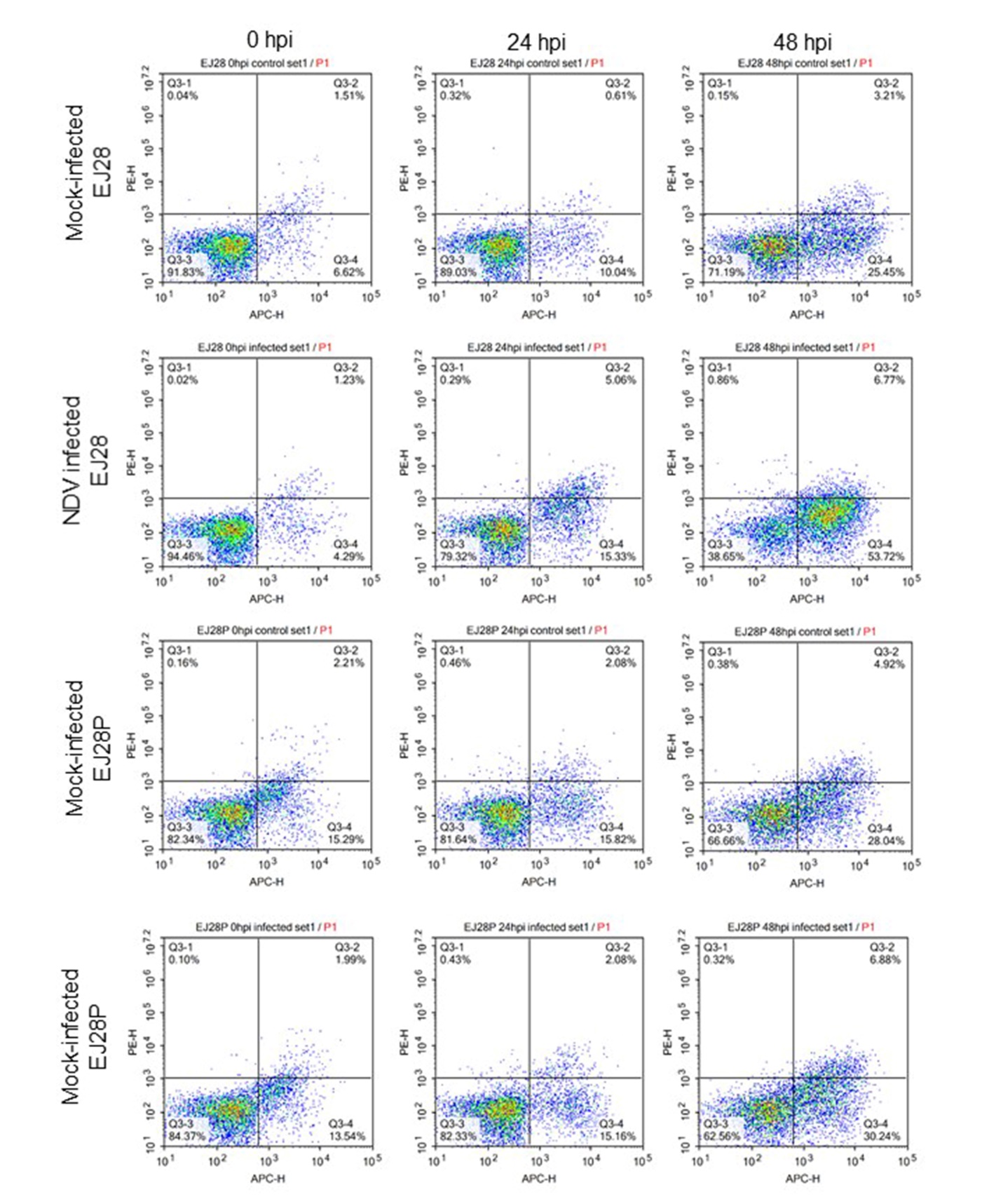
**

**Figure S3**: **Parental EJ28 and EJ28P infected or mock-infected with NDV labelled with annexin V and PI.** Lower left quadrants, viable cells. Lower right quadrants, early apoptotic cells. Upper left quadrants, necrotic cells. Upper right quadrants, late apoptotic cells. A representative of a biological triplicate is presented here.
