# Supplementary material for: Persistent Newcastle disease virus infection in bladder cancer cells is associated with putative pro-survival and anti-viral transcriptomic changes
Source: BMC Cancer. 2021 May 27;21:625. doi: 10.1186/s12885-021-08345-y (PMC8161962; doi:10.1186/s12885-021-08345-y)
Supplement: Supplementary file 3 — Additional file 3: Figure S4: Gene markers for the EJ28P versus EJ28 comparison; Table S2: Details of top 20 significant DEGs between EJ28P and EJ28; Table S3: Gene set of HALLMARK_KRAS_SIGNALING_UP enriched in EJ28P [file 12885_2021_8345_MOESM3_ESM.docx]

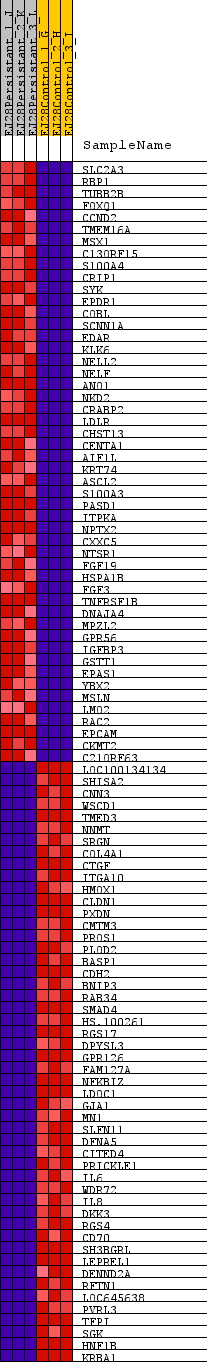

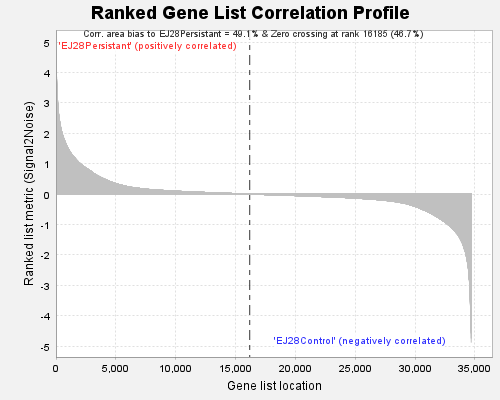


**A**

**B**

**Figure S4: Gene markers for the EJ28P versus EJ28 cells comparison.** (A) Ranked list correlation profile for EJ28P versus EJ28. The dataset has 34694 genes, whereby 16185 genes (46.7%) are positively correlated with EJ28P cells and 18509 genes (53.3%) are negatively correlated with EJ28 cells. (B) Heatmap of the top 50 genes upregulated in EJ28P and EJ28, respectively.

**Table S2: Details of top 20 significant DEGs between EJ28P and EJ28**

| **ILMN_GENE** | **LOG2FC** | **Pval** | **Padj** | **GENE NAME** |
| --- | --- | --- | --- | --- |
| *SLC2A3* | 6.756368 | 1.30E-209 | *4.52E-205* | solute carrier family 2 member 3(SLC2A3) |
| *RBP1* | 6.506529 | 4.21E-193 | 4.86E-189 | retinol binding protein 1(RBP1) |
| *TUBB2B* | 5.700177 | 2.14E-167 | 1.86E-163 | tubulin beta 2B class IIb(TUBB2B) |
| *FOXQ1* | 5.302497 | 1.01E-143 | 6.98E-140 | forkhead box Q1(FOXQ1) |
| *HS.579631 (SNAR-A1)* | 5.798051 | 1.78E-127 | 7.70E-124 | AGENCOURT_10229596 NIH_MGC_141 Homo sapiens cDNA clone IMAGE:6563923 5/ small ILF3/NF90-associated RNA A1(SNAR-A1) |
| *ANO1 (TMEM16A)* | 4.537212 | 1.26E-120 | 4.86E-117 | anoctamin 1(ANO1)/ Homo sapiens transmembrane protein 16A (TMEM16A), mRNA. |
| *MSX1* | 4.466518 | 2.82E-116 | 8.15E-113 | msh homeobox 1(MSX1) |
| *S100A4* | 4.340894 | 3.27E-108 | 6.68E-105 | S100 calcium binding protein A4(S100A4) |
| *CCND2* | 4.598294 | 2.99E-107 | 5.77E-104 | cyclin D2(CCND2) |
| *SYK* | 4.194188 | 3.50E-107 | 6.39E-104 | spleen associated tyrosine kinase(SYK) |
| *C13ORF15 (RGCC)* | 4.418043 | 1.49E-105 | 2.58E-102 | Homo sapiens chromosome 13 open reading frame 15 (C13orf15) / Regulator Of Cell Cycle |
| *LOC100134134* | -6.25003 | 3.79E-195 | 6.57E-191 | PREDICTED: Homo sapiens similar to peroxidasin homolog (LOC100134134), mRNA. |
| *CNN3* | -5.06813 | 1.10E-141 | 6.36E-138 | calponin 3(CNN3) |
| *SHISA2* | -5.52047 | 6.83E-135 | 3.39E-131 | shisa family member 2(SHISA2) |
| *TMED3* | -4.44664 | 9.33E-119 | 3.24E-115 | transmembrane p24 trafficking protein 3(TMED3) |
| *WSCD1* | -4.52059 | 2.79E-118 | 8.79E-115 | WSC domain containing 1(WSCD1) |
| *SRGN* | -4.37329 | 8.29E-115 | 2.18E-111 | serglycin(SRGN) |
| *CTGF* | -4.25848 | 8.81E-115 | 2.18E-111 | connective tissue growth factor(CTGF) |
| *NNMT* | -4.37525 | 3.98E-112 | 9.21E-109 | nicotinamide N-methyltransferase(NNMT) |
| *COL4A1* | -4.37142 | 1.10E-110 | 2.38E-107 | collagen type IV alpha 1 chain(COL4A1) |

**Table S3: Gene set of HALLMARK_KRAS_SIGNALING_UP enriched in EJ28P**

|  | **SYMBOL** | **TITLE** | **RANK IN GENE LIST** | **RANK METRIC SCORE** | **RUNNING ES** | **CORE ENRICHMENT** |
| --- | --- | --- | --- | --- | --- | --- |
| 1 | [CCND2](https://ensembl.org/Search/Results?q=CCND2) | "Homo sapiens cyclin D2 (CCND2), mRNA." | 4 | 4.601 | 0.0296 | Yes |
| 2 | [ANO1](https://ensembl.org/Search/Results?q=ANO1) | "Homo sapiens anoctamin 1, calcium activated chloride channel (ANO1), transcript variant 1, mRNA." | 18 | 4.359 | 0.0575 | Yes |
| 3 | [TNFRSF1B](https://ensembl.org/Search/Results?q=TNFRSF1B) | "Homo sapiens tumor necrosis factor receptor superfamily, member 1B (TNFRSF1B), mRNA." | 36 | 4.012 | 0.0829 | Yes |
| 4 | [MPZL2](https://ensembl.org/Search/Results?q=MPZL2) | "Homo sapiens myelin protein zero-like 2 (MPZL2), transcript variant 2, mRNA." | 38 | 3.976 | 0.1086 | Yes |
| 5 | [IGFBP3](https://ensembl.org/Search/Results?q=IGFBP3) | "Homo sapiens insulin-like growth factor binding protein 3 (IGFBP3), transcript variant 2, mRNA." | 40 | 3.972 | 0.1343 | Yes |
| 6 | [EMP1](https://ensembl.org/Search/Results?q=EMP1) | "Homo sapiens epithelial membrane protein 1 (EMP1), mRNA." | 150 | 2.960 | 0.1503 | Yes |
| 7 | [TMEM158](https://ensembl.org/Search/Results?q=TMEM158) | "Homo sapiens transmembrane protein 158 (TMEM158), mRNA." | 291 | 2.420 | 0.1619 | Yes |
| 8 | [FGF9](https://ensembl.org/Search/Results?q=FGF9) | "Homo sapiens fibroblast growth factor 9 (glia-activating factor) (FGF9), mRNA." | 310 | 2.363 | 0.1766 | Yes |
| 9 | [SEMA3B](https://ensembl.org/Search/Results?q=SEMA3B) | "Homo sapiens sema domain, immunoglobulin domain (Ig), short basic domain, secreted, (semaphorin) 3B (SEMA3B), transcript variant 2, mRNA." | 385 | 2.205 | 0.1888 | Yes |
| 10 | [PCSK1N](https://ensembl.org/Search/Results?q=PCSK1N) | "Homo sapiens proprotein convertase subtilisin/kexin type 1 inhibitor (PCSK1N), mRNA." | 450 | 2.086 | 0.2004 | Yes |
| 11 | [MAFB](https://ensembl.org/Search/Results?q=MAFB) | "Homo sapiens v-maf musculoaponeurotic fibrosarcoma oncogene homolog B (avian) (MAFB), mRNA." | 455 | 2.075 | 0.2137 | Yes |
| 12 | [ETV4](https://ensembl.org/Search/Results?q=ETV4) | "Homo sapiens ets variant 4 (ETV4), transcript variant 1, mRNA." | 490 | 2.027 | 0.2258 | Yes |
| 13 | [PLAUR](https://ensembl.org/Search/Results?q=PLAUR) | "Homo sapiens plasminogen activator, urokinase receptor (PLAUR), transcript variant 1, mRNA." | 499 | 2.011 | 0.2386 | Yes |
| 14 | [MMD](https://ensembl.org/Search/Results?q=MMD) | "Homo sapiens monocyte to macrophage differentiation-associated (MMD), mRNA." | 501 | 2.009 | 0.2516 | Yes |
| 15 | [CSF2](https://ensembl.org/Search/Results?q=CSF2) | "Homo sapiens colony stimulating factor 2 (granulocyte-macrophage) (CSF2), mRNA." | 720 | 1.752 | 0.2566 | Yes |
| 16 | [GADD45G](https://ensembl.org/Search/Results?q=GADD45G) | "Homo sapiens growth arrest and DNA-damage-inducible, gamma (GADD45G), mRNA." | 778 | 1.689 | 0.2659 | Yes |
| 17 | [ID2](https://ensembl.org/Search/Results?q=ID2) | "Homo sapiens inhibitor of DNA binding 2, dominant negative helix-loop-helix protein (ID2), mRNA." | 783 | 1.684 | 0.2766 | Yes |
| 18 | [NRP1](https://ensembl.org/Search/Results?q=NRP1) | "Homo sapiens neuropilin 1 (NRP1), transcript variant 1, mRNA." | 928 | 1.554 | 0.2825 | Yes |
| 19 | [TSPAN13](https://ensembl.org/Search/Results?q=TSPAN13) | "Homo sapiens tetraspanin 13 (TSPAN13), mRNA." | 951 | 1.535 | 0.2918 | Yes |
| 20 | [RGS16](https://ensembl.org/Search/Results?q=RGS16) | "Homo sapiens regulator of G-protein signalling 16 (RGS16), mRNA." | 1267 | 1.326 | 0.2913 | Yes |
| 21 | [CBX8](https://ensembl.org/Search/Results?q=CBX8) | "Homo sapiens chromobox homolog 8 (Pc class homolog, Drosophila) (CBX8), mRNA." | 1301 | 1.305 | 0.2987 | Yes |
| 22 | [DUSP6](https://ensembl.org/Search/Results?q=DUSP6) | "Homo sapiens dual specificity phosphatase 6 (DUSP6), transcript variant 1, mRNA." | 1411 | 1.248 | 0.3037 | Yes |
| 23 | [USP12](https://ensembl.org/Search/Results?q=USP12) | "Homo sapiens ubiquitin specific peptidase 12 (USP12), mRNA." | 1487 | 1.216 | 0.3094 | Yes |
| 24 | [SLPI](https://ensembl.org/Search/Results?q=SLPI) | "Homo sapiens secretory leukocyte peptidase inhibitor (SLPI), mRNA." | 1508 | 1.207 | 0.3166 | Yes |
| 25 | [KCNN4](https://ensembl.org/Search/Results?q=KCNN4) | "Homo sapiens potassium intermediate/small conductance calcium-activated channel, subfamily N, member 4 (KCNN4), mRNA." | 1640 | 1.149 | 0.3202 | Yes |
| 26 | [SNAP25](https://ensembl.org/Search/Results?q=SNAP25) | "Homo sapiens synaptosomal-associated protein, 25kDa (SNAP25), transcript variant 2, mRNA." | 1745 | 1.101 | 0.3243 | Yes |
| 27 | [WDR33](https://ensembl.org/Search/Results?q=WDR33) | "Homo sapiens WD repeat domain 33 (WDR33), transcript variant 3, mRNA." | 1804 | 1.077 | 0.3296 | Yes |
| 28 | [USH1C](https://ensembl.org/Search/Results?q=USH1C) | "Homo sapiens Usher syndrome 1C (autosomal recessive, severe) (USH1C), transcript variant 1, mRNA." | 1811 | 1.074 | 0.3364 | Yes |
| 29 | [F2RL1](https://ensembl.org/Search/Results?q=F2RL1) | "Homo sapiens coagulation factor II (thrombin) receptor-like 1 (F2RL1), mRNA." | 1933 | 1.028 | 0.3395 | Yes |
| 30 | [ALDH1A3](https://ensembl.org/Search/Results?q=ALDH1A3) | "Homo sapiens aldehyde dehydrogenase 1 family, member A3 (ALDH1A3), mRNA." | 1957 | 1.021 | 0.3455 | Yes |
| 31 | [HKDC1](https://ensembl.org/Search/Results?q=HKDC1) | "Homo sapiens hexokinase domain containing 1 (HKDC1), mRNA." | 2050 | 0.986 | 0.3492 | Yes |
| 32 | [TRIB1](https://ensembl.org/Search/Results?q=TRIB1) | "Homo sapiens tribbles homolog 1 (Drosophila) (TRIB1), mRNA." | 2065 | 0.982 | 0.3551 | Yes |
| 33 | [SERPINA3](https://ensembl.org/Search/Results?q=SERPINA3) | "Homo sapiens serpin peptidase inhibitor, clade A (alpha-1 antiproteinase, antitrypsin), member 3 (SERPINA3), mRNA." | 2159 | 0.953 | 0.3586 | Yes |
| 34 | [ABCB1](https://ensembl.org/Search/Results?q=ABCB1) | "Homo sapiens ATP-binding cassette, sub-family B (MDR/TAP), member 1 (ABCB1), mRNA." | 2330 | 0.902 | 0.3595 | Yes |
| 35 | [ETV5](https://ensembl.org/Search/Results?q=ETV5) | "Homo sapiens ets variant gene 5 (ets-related molecule) (ETV5), mRNA." | 2403 | 0.881 | 0.3631 | Yes |
| 36 | [AVL9](https://ensembl.org/Search/Results?q=AVL9) | "Homo sapiens AVL9 homolog (S. cerevisiase) (AVL9), mRNA." | 2544 | 0.842 | 0.3645 | Yes |
| 37 | [GPNMB](https://ensembl.org/Search/Results?q=GPNMB) | "Homo sapiens glycoprotein (transmembrane) nmb (GPNMB), transcript variant 2, mRNA." | 2593 | 0.830 | 0.3685 | Yes |
| 38 | [PLEK2](https://ensembl.org/Search/Results?q=PLEK2) | "Homo sapiens pleckstrin 2 (PLEK2), mRNA." | 2597 | 0.829 | 0.3737 | Yes |
| 39 | [MMP9](https://ensembl.org/Search/Results?q=MMP9) | "Homo sapiens matrix metallopeptidase 9 (gelatinase B, 92kDa gelatinase, 92kDa type IV collagenase) (MMP9), mRNA." | 2628 | 0.820 | 0.3782 | Yes |
